# Hierarchical Motion Field Alignment for Robust Optical Flow Estimation

**DOI:** 10.3390/s25092653

**Published:** 2025-04-22

**Authors:** Dianbo Ma, Kousuke Imamura, Ziyan Gao, Xiangjie Wang, Satoshi Yamane

**Affiliations:** 1 Graduate School of Natural Science and Technology, Kanazawa University, Kanazawa 9201192, Japan; imamura@ec.t.kanazawa-u.ac.jp (K.I.); syamane@is.t.kanazawa-u.ac.jp (S.Y.); 2 School of Information Science, Japan Advanced Institute of Science and Technology, Nomi 9231292, Japan; ziyan-g@jaist.ac.jp; 3 State Key Laboratory of Automotive Simulation and Control, Jilin University, Changchun 130022, China; xiangjie18@mails.jlu.edu.cn

**Keywords:** attention mechanisms, computer vision, correlation, deep learning, image processing, motion estimation, optical flow, recurrent neural networks, residual neural networks, supervised learning

## Abstract

Optical flow estimation is a fundamental and long-standing task in computer vision, facilitating the understanding of motion within visual scenes. In this study, we aim to improve optical flow estimation, particularly in challenging scenarios involving small and fast-moving objects. Specifically, we proposed a learning-based model incorporating two key components: the Hierarchical Motion Field Alignment module, which ensures accurate estimation of objects of varying sizes while maintaining manageable computational complexity, and the Correlation Self-Attention module, which effectively handles large displacements, making the model suitable for scenarios with fast-moving objects. Additionally, we introduced a Multi-Scale Correlation Search layer to enhance the four-dimensional cost volume, enabling the model to address various types of motion. Experimental results demonstrate that our model achieves superior generalization performance and significantly improves the estimation of small, fast-moving objects.

## 1. Introduction

Optical flow estimates dense two-dimensional (2D), per-pixel motion by identifying the most correlated pixels between consecutive frames in a video sequence. It is a fundamental yet challenging task in computer vision and has been widely applied to various downstream tasks, including video surveillance [1,2], video editing [3,4], image stitching [5,6], action recognition [7], robot navigation [8], visual SLAM [9,10], visual tracking [11,12,13], and autonomous driving [14]. Early variational methods [15,16,17] were introduced for optical flow estimation, paving the way for more advanced algorithms [18,19,20] and subsequent studies. However, constrained by handcrafted features, these traditional methods struggle to capture large displacements and handle complex motion patterns, particularly in dynamic and rapidly changing environments.

Recent advancements in deep convolutional neural networks (CNNs) have enabled learning-based methods [21,22,23,24,25,26,27,28,29,30] to surpass traditional energy optimization approaches for optical flow estimation. FlowNet [21] was the first to achieve state-of-the-art performance by directly regressing optical flow within an end-to-end learning framework. Building on this foundation, PWC-Net [22] introduced a coarse-to-fine structure that computes and preserves pixel-wise feature correspondences, inspiring several enhanced and lightweight variants [25,28,31,32]. Unrolled and iterative refinement designs have further improved the accuracy of optical flow estimation. RAFT (Recurrent All-Pairs Field Transforms) [26] is a representative example of this approach, setting a new benchmark in the field. It learns similarity matching between all pixel pairs by constructing multi-scale, four-dimensional (4D) cost volumes. A gated recurrent unit (GRU) [33] is then used to iteratively refine and regress optical flow by referencing motion features within the cost volumes. Subsequently, several methods [29,34,35,36,37,38,39] have been developed to further enhance optical flow precision. To address memory constraints, some approaches [34,35,36] employ sparse strategies or decoupling techniques when computing cost volumes, achieving more efficient inference at the expense of some accuracy. In contrast, our method reduces the computational burden by downsampling hierarchical motion features through convolution operations, followed by motion feature aggregation.

Unlike traditional CNNs, vision transformers (ViTs) [40,41] more effectively capture global dependencies that are essential for identifying optimal motion representations necessary for accurate flow field estimation. Attention mechanisms have been widely employed to address challenges such as occlusions, large displacements, and high computational cost [29,34,35,37]. For instance, global motion aggregation (GMA) [29] uses attention mechanisms to aggregate precise motion features from non-occluded regions and uses them as guidance for flow prediction in occluded areas. Leveraging the low-pass characteristics of ViTs, CRAFT [37] introduced a semantic smoothing layer and a cross-attention layer to improve contextual feature fusion and enhance standard correlation volumes, respectively. Some of these methods use Vision Transformers (ViTs) for feature extraction, apply cross-attention to construct cost volumes, or model spatiotemporal dependencies. However, they often suffer from low inference efficiency. In contrast, we employ a lightweight attention block to model global motion associations, ensuring high inference efficiency without sacrificing accuracy.

**Figure 1:**
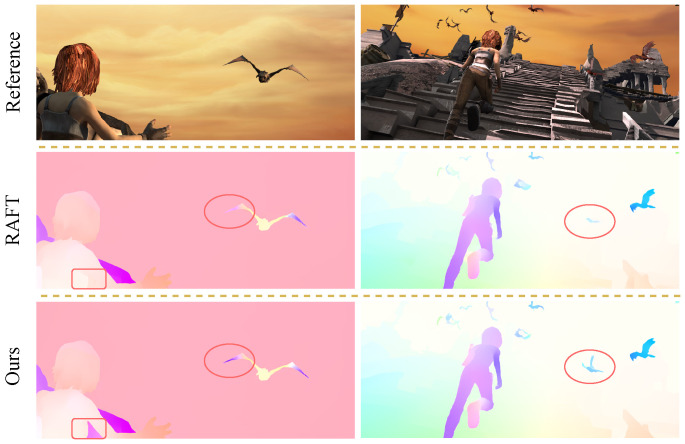
Visual comparisons between the proposed model and RAFT [26] on the Sintel [42] dataset. First, second, and third rows show the input images, RAFT model’s results, and proposed model’s results, respectively. Performance differences are highlighted using red circles and squares, drawing attention to areas where the two methods diverge. The proposed model provides more accurate estimates for small objects and sharp edges, showcasing the effectiveness of newly introduced modules.

Existing methods struggle with small, fast-moving objects, particularly when high-resolution inputs are downsampled, as this leads to ambiguities and errors in both cost volume generation and flow field refinement. To address this issue, we developed a new model based on the RAFT framework, termed the Hierarchical Motion Field Alignment Flow model (HMAFlow). This novel optical flow framework incorporates two key components: the Hierarchical Motion Field Alignment (HMA) module, which effectively integrates multi-level motion features into a unified, high-quality cost volume—enabling the model to handle objects of various sizes while maintaining manageable computational complexity—and the Correlation Self-Attention (CSA) module, which employs a self-attention mechanism to further refine the cost volume by capturing more accurate global motion associations, thereby ensuring reliable optical flow estimation in scenarios involving large displacements. In addition, we reformulate the conventional 4D cost volumes by computing feature similarities across all pixel pairs at each level of the corresponding feature maps. Unlike RAFT, which applies average pooling to the initial matching matrix to generate 4D pyramidal cost volumes, we introduce a Multi-Scale Correlation Search (MCS) layer that dynamically retrieves motion features using multiple search thresholds from hierarchical feature matching matrices while iteratively refining flow predictions. The cost volumes constructed by the MCS layer are capable of handling diverse motion scenarios, including small motions of small objects, large motions of small objects, small motions of large objects, and large motions of large objects. These advanced modules empower HMAFlow to accurately capture fine details of small targets (see Figure 1).

HMAFlow was evaluated on leading optical flow benchmarks, demonstrating superior cross-dataset generalization performance compared to existing methods [26,29,37,39,43,44], particularly on the Sintel [42] (clean) benchmark. On the KITTI 2015 [45] test set, HMAFlow delivered competitive results, outperforming several high-performing algorithms [26,29,37,43,46]. The contributions of this study are as follows:

- Novel Framework Design: The goal of our research is to improve optical flow algorithms rather than to explore the application of a specific method within a particular domain. To this end, we developed a novel optical flow framework, HMAFlow, aimed at enhancing the accuracy of optical flow estimation, especially for small and fast-moving objects.
- Innovative Modules: HMAFlow incorporates two new modules: the HMA module, which aggregates hierarchical motion features into a unified cost volume, enabling the model to handle objects of various sizes while maintaining manageable computational complexity; and the CSA module, which captures global motion associations to ensure reliable optical flow estimation in scenarios with large displacements.
- Enhanced MCS Layer: We introduced an enhanced Multi-Scale Correlation Search (MCS) layer to construct multi-level cost volumes. This layer incorporates motion features at different resolutions with varying receptive fields, enabling the model to handle motion targets of various sizes and diverse motion scenarios effectively.
- Performance Improvements: Extensive experiments on major benchmarks demonstrated the effectiveness of HMAFlow. Compared to the baseline RAFT, HMAFlow reduced the end-point error (EPE) by 14.2% on the Sintel (clean pass) benchmark and improved the Fl-all metric by 6.8% on the KITTI benchmark.

## 2. Related Work

### 2.1. Optimization-Based Methods

Optical flow estimation from pairs of consecutive video frames is a fundamental task in computer vision. Early methods [15,16,17,47,48,49,50] formulated optical flow estimation as an energy minimization problem by optimizing a set of well-defined objective functions. Subsequently, several studies reformulated optical flow prediction using discrete and global optimization strategies, such as discrete inference in conditional random fields (CRFs) [51], global optimization techniques [52], and regression over four-dimensional (4D) correlation volumes [53]. Another important research direction focused on enhancing feature matching [54] and enforcing motion smoothness [55,56], under the key assumption of brightness constancy. However, these approaches failed to accurately capture small targets, handle large motions, and preserve the intricate details present in complex, real-world scenarios.

### 2.2. Learning-Based Methods

Deep learning has helped address challenges in various visual tasks, and continuous advancements in deep learning techniques have significantly improved the accuracy of optical flow estimation. For instance, FlowNet [21] was the first model developed to predict optical flow within an end-to-end framework, where learned deep features were used to compute motion patterns and infer the flow field. Since then, several learning-based methods [22,23,24,25,26,27,57] have been proposed to further enhance optical flow prediction. FlowNet2.0 [23] incorporated multiple stacked flow prediction modules arranged in a coarse-to-fine manner, which iteratively refined the final flow estimation. PWC-Net [22] leveraged pyramidal feature extraction and warping operations to construct a cost volume, which was then processed by a multi-layer CNN to predict optical flow—thereby improving performance while reducing model complexity.

RAFT [26] stands out as a notable representative. It constructs four-dimensional (4D) all-pairs cost volumes to store feature correspondences, upon which a refinement layer iteratively performs lookup operations to obtain the desired flow estimation. Building upon RAFT’s structural design, numerous subsequent studies [29,34,35,36,37,58] have explored methods to further improve the accuracy and stability of optical flow estimation. For instance, SCV [36] introduced a sparse cost volume by calculating k-nearest matches as a substitute for dense displacement representations, which significantly reduced both computational cost and memory usage. Similarly, Separable Flow [35] decomposed the cost volume computation into a series of one-dimensional (1D) operations, effectively lowering computational complexity and memory footprint. Although these methods achieved reduced computational overhead, their overall performance often remained suboptimal. Optical flow estimation has also been revisited from the perspectives of training strategies and data augmentation, leading to improvements in both accuracy and efficiency over existing techniques [38,59,60]. SOFIA [61] presented a novel multi-scale optical flow reconstruction algorithm that leverages spectral and structural information from color images to enhance accuracy and mitigate the ill-posed nature of inverse problems. EMD-S [44] proposed an efficient optical flow framework comprising a multi-scale motion aggregation module and a confidence-induced flow propagation module. While the multi-scale module in EMD-S employs a selective strategy to fuse flow fields, our proposed approach utilizes hierarchical motion field alignment to integrate multi-level motion features.

### 2.3. Attention Mechanism in Optical Flow

ViTs [40] can efficiently learn long-range dependencies; therefore, attention mechanisms have been employed to enhance feature representations and enable global matching between image pairs [29,34,37,39,46,58,62]. These mechanisms are particularly useful for handling occlusions and capturing large displacements in complex scenarios involving small targets and challenging noise. Building upon the RAFT framework, GMA [29] introduced a global motion aggregation module, which improved optical flow modeling in occluded regions. Flow1D [34] decoupled two-dimensional (2D) correspondence into separate one-dimensional (1D) attention and correlation operations for large-displacement matching in high-resolution images; these operations were applied independently along the vertical and horizontal directions. FlowFormer [58] adopted a fully transformer-based architecture to restructure the conventional refinement pipeline. It utilized alternating group transformer layers to encode the 4D cost volume, while recurrent vision transformer blocks decoded the cost memory to generate more accurate flow predictions. Moreover, explicit or global matching techniques were employed to address challenges posed by large displacements and complex motion patterns [46,62]; these approaches significantly improved the efficiency and quality of optical flow inference. Despite their strong performance across multiple benchmarks, these methods heavily rely on attention modules, resulting in high computational costs and increased inference time.

## 3. Proposed Method

**Figure 2:**
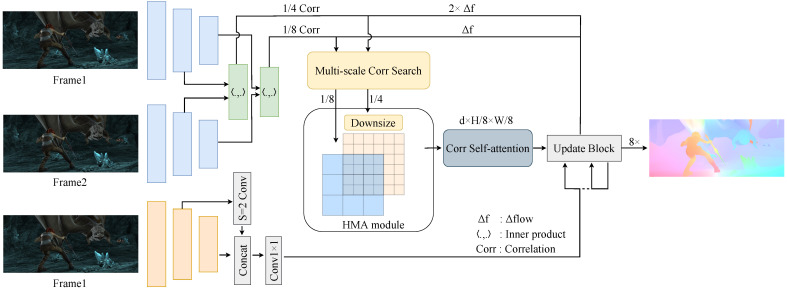
Overall framework of the proposed HMAFlow. It introduces two new modules: (1) the Hierarchical Motion Field Alignment (HMA) module and (2) the Correlation Self-Attention (CSA) module. Furthermore, we develop a Multi-Scale Correlation Search (MCS) layer, which enhances the original 4D cost volume by extending it into two levels of multi-scale cost volumes, with four layers at each level. The feature encoder and context encoder both consist of six residual blocks. To integrate multi-scale contextual features, a skip connection layer is added to the context network. For the optical flow regressor, we follow the baseline RAFT [26] and utilize a convolutional Gated Recurrent Unit (GRU [33]) network to refine the predicted optical flow.

HMAFlow, a novel and highly effective model, was designed for optical flow estimation; its architecture is shown in Figure 2. The core structure of HMAFlow comprises the HMA module, which unifies motion features across different scales, and the CSA module, which enhances global motion features. The model also integrates an improved MCS layer for handling complex and diverse motion patterns. The proposed model and its components are comprehensively discussed in subsequent sections.

### 3.1. Preliminaries

For a pair of consecutive input images, $I_{1}$ and $I_{2}\in R^{H\times W\times 3}$, optical flow methods estimate the 2D displacement field, $f=\left(f_{u},f_{v}\right)\in R^{H\times W\times 2}$. This field accurately maps the coordinates p=(x,y) of each pixel in $I_{1}$ to its corresponding pixel $p^{\prime }=\left(x+f_{u}\left(x\right),y+f_{v}\left(y\right)\right)$ in $I_{2}$. In a standard optical flow pipeline, such as RAFT [26], weight-sharing feature encoders are employed to extract high-quality feature representations, $F_{1}$ and $F_{2}\in R^{H^{\prime }\times W^{\prime }\times D}$, from both images. These feature maps have dimensions $H^{\prime }$, $W^{\prime }$, and *D*, which represent the height, width, and depth (or number of channels) of downsampled features, respectively. A context extraction network is used for exclusively learning contextual features, ${F_{1}}^{c}\in R^{H^{\prime }\times W^{\prime }\times D}$, from image $I_{1}$. These features are subsequently fed into a convolutional refinement network, typically a GRU [33], to refine flow estimation.

The success of the iterative refinement paradigm relies heavily on dense 4D correlation volumes. These 4D pyramidal volumes ($H^{\prime }\times W^{\prime }\times H^{\prime }/2^{k}\times W^{\prime }/2^{k}$) are constructed by calculating the inner product between all vector pairs from $F_{1}$ and $F_{2}$, generating an initial correlation volume. Subsequently, average pooling is applied to the last two dimensions at multiple scales {1,2,4,8} to create a multi-scale representation. Finally, the convolutional refinement network iteratively queries these correlation features, alongside the contextual features, to progressively regress and update the estimated flow field.

### 3.2. Multi-Scale Cost Volumes

**Figure 3:**
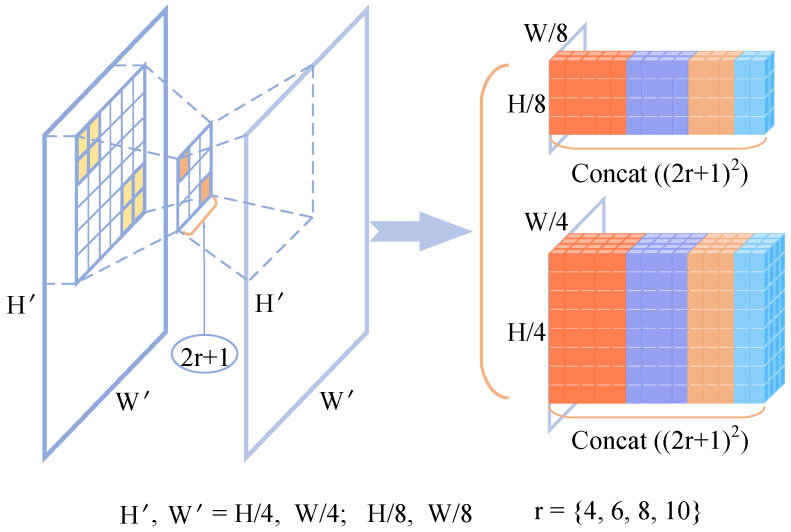
The illustration presents the Multi-Scale Correlation Search strategy, where we apply multiple search ranges to independently conduct lookup operations on each of the two-level base 4D cost volumes. For each level, this process generates a 3D pyramid-shaped cost volume, enabling more precise and efficient motion estimation across different scales.

**Figure 4:**
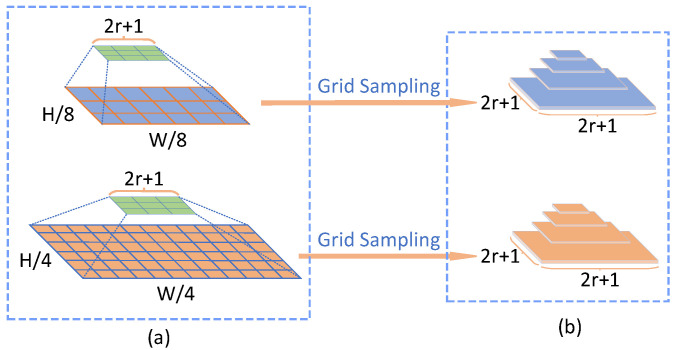
Grid sampling details. (**a**) illustrates the sampling of each 2D correlation feature map from the base cost volumes at 1/4 and 1/8 resolutions using a sampling grid with a radius of *r*, repeated four times. (**b**) shows that after sampling, each 2D correlation feature map generates a four-level correlation feature pyramid, with each level having dimensions of (2r+1)×(2r+1).

Feature extraction. The feature and context encoders utilized in our model maintain the same structural design as those found in RAFT [26]. While it is possible to employ more feature maps at different resolutions during the construction of cost volumes, we limit our approach to the output feature maps from two resolution scales, considering the trade-off between computational complexity and correlation reliability. Specifically, the feature extraction process can be expressed as
  (1) $$g_{\theta }^{\;l}\left(I_{1},I_{2}\right)\mapsto \left\{F_{1}^{l},F_{2}^{l}\right\},\;\;F_{i}^{l}\in R^{lH\times lW\times D}$$
  where *g* represents the feature encoder with parameters θ, *l* corresponds to the output layers at 1/4 and 1/8 resolution, and the feature dimension D is set to 384. Notably, both output layers produce feature maps with an identical number of channels. Additionally, we utilize output features at the same resolution from the context network $h_{\theta }$ and apply a skip connection to merge these contextual features.
Correlation computation. For each feature vector in $F_{1}^{l}$, a corresponding 2D correlation map is computed by comparing it with every feature vector in $F_{2}^{l}$. The standard cost volume is constructed by calculating the inner product between all possible pairs of feature vectors from $F_{1}^{l}$ and $F_{2}^{l}$, effectively capturing their correlations. We formulate the construction of 4D cost volumes as
  (2) $$\begin{array}{cc} \; & C\left(g_{\theta }^{\;l}\left(I_{1}\right),g_{\theta }^{\;l}\left(I_{2}\right)\right)\in R^{lH\times lW\times lH\times lW}\\ \; & C_{ijmn}^{l}=\sum_{h}g_{\theta }^{\;l}{\left(I_{1}\right)}_{ijh}\;\cdot \;g_{\theta }^{\;l}{\left(I_{2}\right)}_{mnh}\\ \; & C^{l}=Set\left(C_{ijmn}^{l}\right) \end{array}$$
  where we denote the base volume (a 4D volume) as $C^{l}$, *l* represents the resolution level of the feature maps, and Set(·) denotes the set of motion features at the l-resolution layer. The process ensures that motion information at multiple scales can be preserved, enabling more accurate flow estimation.
Multi-scale search. Unlike RAFT [26], which applies an average pooling operation on the last two dimensions of the original volume, our approach utilizes multiple search ranges to iteratively query the primary hierarchical volume, thereby generating multi-scale cost volumes. The hierarchically multi-scale cost volumes, denoted as $\left\{C_{1∼4}^{1/4},C_{5∼8}^{1/8}\right\}$, comprise two distinct levels, each represented by a 4-layer pyramid structure. The construction process of multi-scale cost volumes can be seen in Figure 3 and Figure 4. The correlation pyramid at the 1/4 resolution effectively captures both subtle and extensive movements of small objects, while the pyramid at the 1/8 resolution is proficient at detecting a broader range of motions in larger targets.

We enhance the lookup operator utilized in RAFT by implementing multiple neighborhood searches, which results in four sampled maps corresponding to each 2D correlation map within the 4D base volume at *l* resolution. Based on the aforementioned optical flow definition, we can define multi-scale local neighborhoods with a radius $r_{i}\in \left\{4,6,8,10\right\}$ around $p^{\prime }$

(3) $$\begin{array}{c} N_{r_{i}}\left(p^{\prime }\right)=\{p^{\prime }+\delta \;|\;\delta \in Z^{2}{,||\delta ||}_{\infty }\leq r_{i}\} \end{array}$$

to sample features from the correlation volumes. It is important to note that we advocate for the use of $L_{\infty }$ (Chebyshev distance) in defining these local neighborhoods. This multi-scale search strategy is then applied to the two primary volumes, resulting in two levels of 4-layer pyramidal correlation volumes. The sampled features from each 4-layer pyramid at both levels are concatenated into a single 3D volume, as illustrated in Figure 3. Therefore, our multi-scale search and cost volumes can be represented as

(4) $$\begin{array}{cc} \; & S\left(r_{i},C^{l}\right)\in R^{lH\times lW\times {\left(2r_{i}+1\right)}^{2}\times {\left(2r_{i}+1\right)}^{2}}\\ \; & M^{l}\left(C\left(g_{\theta }^{\;l}\left(I_{1}\right),g_{\theta }^{\;l}\left(I_{2}\right)\right)\right)=Concat\left(S\left(r_{i},C^{l}\right)\right) \end{array}$$

where S(·,·) signifies the search operator and $M^{l}$ denotes each level of the 4-layer cost volumes.

### 3.3. Hierarchical Motion Field Alignment

Each feature vector in $F_{1}^{l}$ generates a corresponding 2D response map, which shares the same height lH and width lW as $F_{2}^{l}$. After sampling the 4D cost volumes, each 2D response map is compressed into a vector of length $d=\sum {\left(2r_{i}+1\right)}^{2}$, effectively transforming the two levels of 4D cost volumes into two levels of 3D cost volumes. These two levels of 3D cost volumes differ in their height lH and width lW but maintain the same feature dimension *d*, as shown in Figure 3. Within a 3D volume, a 2D plane along the height and width directions contains a set of motion features sampled with radius $r_{i}$ from a region of consistent location and size across all 2D response maps in the 4D volume. Moreover, a vector along the *d*-dimension in a 3D volume represents a set of global motion features, sampled with four radii from the 2D response map, which is produced by calculating the correlation between a feature vector at the same location in $F_{1}^{l}$ and all feature vectors in $F_{2}^{l}$.

**Figure 5:**
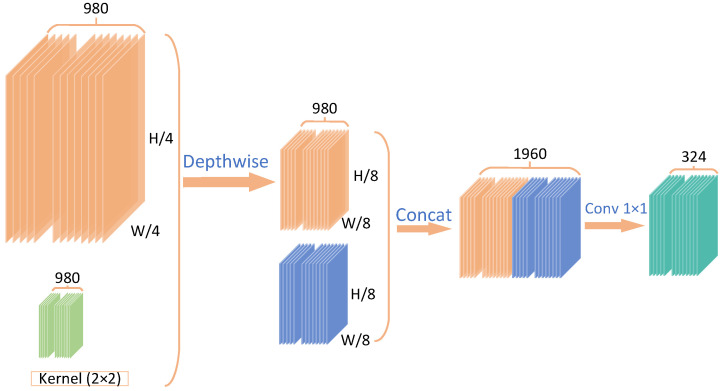
Details of the HMA module. This module applies a depthwise convolution with a kernel size of 2×2 to the 1/4-resolution multi-scale cost volumes, producing 1/8-resolution cost volumes. Subsequently, two 1/8-resolution cost volumes are concatenated, followed by a dimensionality reduction operation, resulting in 1/8-resolution cost volumes with 324 channels.

Based on these observations, we infer that a 2×2 region in the 2D plane along the height and width directions of $M^{1/4}$ and a 1×1 region in the corresponding position of the 2D plane along the height and width directions of $M^{1/8}$ should contain equivalent information, as they share the same contextual receptive field. Therefore, we introduce the Hierarchical Motion Field Alignment (HMA) module, designed to merge the two levels of 3D cost volumes (see Figure 5 for details). The HMA module consists of a 2×2 convolutional layer followed by a ReLU activation and a 1×1 convolutional layer, also followed by ReLU. We first apply a 2×2 depthwise convolution with a stride of 2 on the 3D cost volume $M^{1/4}$, which reduces its resolution to match that of $M^{1/8}$. Next, the two 3D cost volumes with the same dimensions are concatenated along the *d*-axis to form a single 3D cost volume. This volume is then passed through the 1×1 convolutional layer for dimensionality reduction. Ultimately, the HMA module produces a high-quality global cost volume with dimensions of H/8×W/8×324. We formally define the entire process as follows:

(5) $$\begin{array}{cc} \; & A\left(M^{1/4},M^{1/8}\right)=Concat\left(Conv_{2\times 2}\left(M^{1/4}\right),M^{1/8}\right)\\ \; & DR\left(A^{*}\right)=Conv_{1\times 1}\left(A\left(M^{1/4},M^{1/8}\right)\right) \end{array}$$

where A(·,·) represents the alignment operation, $A^{*}$ denotes the aligned correlation volume, and DR(·) stands for the dimensionality reduction operation.

### 3.4. Self-Attention for Correlation

**Figure 6:**
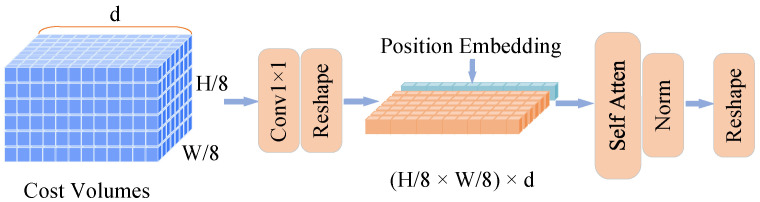
Structure of the Correlation Self-Attention module. After the alignment process, the 3D cost volume at a 1/8 resolution is input into the Correlation Self-Attention (CSA) module. Within the CSA module, we utilize a single optimized attention block, as the high quality of the input 3D volume makes this configuration sufficient to fulfill the model’s requirements. This approach effectively balances performance with computational cost.

Several methods have explored various attention mechanisms applied to cost volumes, demonstrating the significant advantages of attention techniques in acquiring robust global motion features. For example, CRAFT [37] introduced a cross-frame attention module designed to generate the correlation volume between the reference and target frames. Similarly, GMA [29] utilized attention principles to construct a global motion aggregation module, which was employed to aggregate both 2D context features and 2D motion features. However, unlike these approaches, we propose a lightweight Correlation Self-Attention (CSA) module that is specifically designed to further enhance global motion features within a 3D cost volume. Notably, we adapt a current large-scale vision transformer model into a single attention module to better suit the specific requirements of our framework. The detailed structure of the CSA module is illustrated in Figure 6.

We input the 3D cost volume from the HMA module—sized 324×H/8×W/8—into the CSA module to learn global associations between motion features. These associations are computed both within the same cost plane (i.e., along the height and width) and across the *d* dimension. A 1×1 convolution is first applied to the cost volume. Since each 2D plane along the height and width represents responses from all vectors in $F_{1}^{l}$ to a local region in $F_{2}^{l}$, we flatten these planes and reshape the 3D volume into a 2D correlation matrix of size (H/8×W/8,1,324). A global positional embedding is then added to support robust motion modeling. This embedded 2D cost is processed by a single self-attention block to compute reliable correlations. The lightweight CSA module uses only one attention head and two MLPs, achieving efficient optical flow inference with better performance than methods that rely on full ViT architectures or multiple attention layers.

### 3.5. Training Loss

In our approach, we strictly adhere to the original configuration of the objective function as defined in RAFT [26]. Throughout the training and inference process, the model iteratively refines the predicted optical flow. Specifically, the entire training process of our model is supervised by calculating the $L_{1}$ distance between the estimated optical flow and the ground truth flow across the full sequence of predictions, denoted as $\left\{f_{1},f_{2},\ldots ,f_{N}\right\}$. These predictions are weighted by exponentially increasing factors to progressively emphasize later predictions in the sequence. To be more precise, let the ground truth flow be represented as $f_{gt}$; the corresponding supervision loss function can be formulated as

(6) $$\begin{array}{c} L=\sum_{i=1}^{N}\gamma^{N-i}\left|\right|f_{gt}-f_{i}{\left|\right|}_{1} \end{array}$$

where the exponential decay factor, denoted as γ, is set to 0.8 in our experimental settings.

## 4. Experiments

In this section, we present comprehensive experimental results on several standard benchmarks, comparing HMAFlow with recent state-of-the-art methods. We also conduct a thorough ablation study to assess the contributions of individual components. HMAFlow achieves state-of-the-art performance on the Sintel [42] leaderboard with a notable 14.2% reduction in End-Point Error (EPE) on the clean pass subset. On the KITTI 2015 [45] benchmark, it achieves a 6.8% improvement in the Fl-all metric. Moreover, HMAFlow consistently demonstrates superior generalization performance on both datasets. To facilitate future research, we have made the code available at https://github.com/BooTurbo/HMAFlow (accessed on 2 February 2025).

### 4.1. Datasets and Implementation Details

Training schedule. Following previous works [26,29], we first pretrain the model on the FlyingChairs [21] dataset for 120 k iterations with a batch size of 12. We then continue pretraining on FlyingThings [63] for 150 k iterations using a batch size of 6, a process referred to as ‘C + T’. To evaluate generalization, the pretrained model is tested on the training splits of Sintel [42] and KITTI-2015 [45]. For evaluation on the Sintel test set, we finetune the model on a combined dataset consisting of FlyingThings, Sintel, KITTI-2015, and HD1K [64] for 150k iterations (batch size of 6), denoted as ‘C + T + S + K + H’, and submit the results to the Sintel evaluation server. For the KITTI-2015 test set submission, we perform additional finetuning on its training set for 60k iterations with the same batch size.
Evaluation metrics. The Sintel benchmark uses the Average End-Point Error (EPE) as the evaluation metric for optical flow, calculating the average flow error across all pixels. Similarly, the KITTI 2015 benchmark adopts the Fl-all (%) metric, which measures the percentage of outliers (pixels where the flow error exceeds 3 pixels or 5% of the ground truth flow), averaged over all ground truth pixels. For both benchmarks, we report the EPE, with the Fl-all (%) score additionally used for the KITTI evaluation.
Implementation. All experiments related to HMAFlow are implemented using PyTorch 2.6.0 [65]. During both the pretraining and finetuning stages, we apply the AdamW optimizer [66] along with the one-cycle learning rate policy [67]. Our convolutional feature extraction networks follow the RAFT architecture, with the primary modification being an increase in the final feature dimension from 256 to 384. We evaluate various methods on the Sintel and KITTI benchmarks, where our model achieves significantly higher accuracy than other approaches, particularly in handling small targets and large motions, as demonstrated on the public leaderboards.

In our ablation experiments, we train all comparative models exclusively on the FlyingChairs and FlyingThings (C+T) datasets, using the same number of iterations, initial learning rates, and batch sizes as in the generalization training stage. After training, we evaluate the sub-models on the training sets of Sintel and KITTI 2015, recording the EPE and Fl-all results for each experiment.

### 4.2. Comparison with State-of-the-Art Methods

Generalization performance. We present the evaluation results of HMAFlow and other state-of-the-art methods in Table 1. To assess the generalization ability of HMAFlow, we follow previous studies [26,29] by training it on the FlyingChairs and FlyingThings datasets and then evaluating its performance on the Sintel and KITTI training sets. As shown in Table 1, HMAFlow achieves state-of-the-art performance on both the clean and final passes of the Sintel dataset and ranks second on the KITTI 2015 dataset across both key metrics. Specifically, our model records an EPE of 1.24 and 2.47 on the Sintel clean and final passes, respectively. On the KITTI 2015 training set, HMAFlow achieves an EPE of 4.38 and an Fl-all score of 14.90%, which are highly competitive, showing 13.0% and 14.3% improvements over the baseline RAFT. These results clearly demonstrate that HMAFlow exhibits a superior generalization capability compared to RAFT and other methods [29,37,43]. Given that HMAFlow and RAFT share an almost identical refinement stage, we attribute this significant improvement in generalization to the novel modules we introduced.

**Table 1:** Comparisons of various methods in terms of generalization performance. The evaluation metrics include the EPE and the Fl-all metric, where lower values indicate better performance. Following prior works, we evaluate our model on the Sintel [42] and KITTI 2015 [45] training sets after pretraining on the FlyingChairs [21] and FlyingThings [63] datasets. The notation “C + T” refers to the models that were pretrained on these datasets. For clearer comparison, the best results are highlighted in bold.

Training	Method	Sintel (Train) ↓		KITTI-15 (Train) ↓	
		Clean	Final	EPE	Fl-All (%)
C + T	FlowNet2 [23]	2.02	3.54	10.08	30.0
	LiteFlowNet [57]	2.48	4.04	10.39	28.5
	PWC-Net [22]	2.55	3.93	10.35	33.7
	VCN [25]	2.21	3.68	8.36	25.1
	HD3 [68]	3.84	8.77	13.17	24.0
	MaskFlowNet [27]	2.25	3.61	-	23.1
	LiteFlowNet2 [28]	2.24	3.78	8.97	25.9
	DICL-Flow [69]	1.94	3.77	8.70	23.60
	RAFT [26]	1.43	2.71	5.04	17.4
	Flow1D [34]	1.98	3.27	6.69	22.95
	SCV [36]	1.29	2.95	6.80	19.3
	GMA [29]	1.30	2.74	4.69	17.1
	Separable Flow [35]	1.30	2.59	4.60	15.9
	OCTC [38]	1.31	2.67	4.72	16.3
	EMD-S [44]	1.31	2.67	5.00	17.0
	KPA-Flow [39]	1.28	2.68	4.46	15.9
	CRAFT [37]	1.27	2.79	4.88	17.5
	AGFlow [43]	1.31	2.69	4.82	17.0
	DIP [30]	1.30	2.82	**4.29**	**13.73**
	**Ours**	**1.24**	**2.47**	4.38	14.90

**Figure 7:**
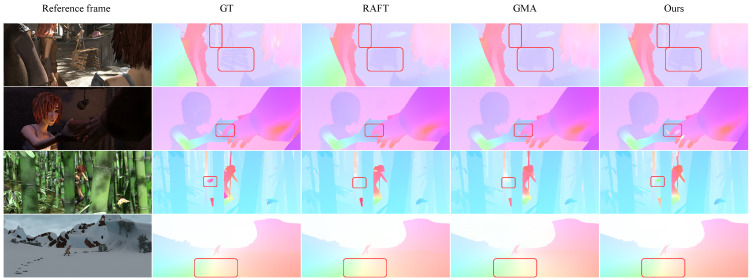
Visual comparisons on the Sintel [42] online benchmark. We compare the proposed HMAFlow model with two well-known algorithms, RAFT [26] and GMA [29]. From left to right, each column shows the input image, ground truth, RAFT inference, GMA inference, and the inference result from our model, respectively. To facilitate a more intuitive comparison, the performance differences among the evaluated methods are highlighted using red rectangular boxes. As illustrated in the figure, our model demonstrates superior performance in accurately detecting small objects, clearly delineating boundaries between different objects, and producing more precise and robust optical flow estimations. In contrast, RAFT and GMA often blur object boundaries and, in many cases, fail to recover fine details, particularly when handling smaller objects.

Sintel benchmark. For the Sintel online testing, we adopt the widely used warm-start strategy for flow inference, following established practices from previous studies [26,29,37]. This strategy enables the model to use previously predicted flow as an initial estimate, thereby improving the accuracy of subsequent predictions. The middle two columns of Table 2 present a detailed quantitative comparison of results on the Sintel benchmark, where our proposed method, HMAFlow, achieves the best End-Point Error (EPE) score of 1.38 among all state-of-the-art approaches on the clean pass. Although our method does not achieve the top performance on the final pass, it still delivers results comparable to the leading methods [26,30,62] in the field. To further demonstrate the effectiveness of HMAFlow, we compare it with the baseline RAFT [26] and GMA [29] models on the Sintel test set, and visual comparisons of their flow predictions are shown in Figure 7. Notably, HMAFlow provides more precise flow predictions, especially for fine contours, detailed structures, and object boundaries, outperforming both RAFT and GMA in these areas.

**Table 2:** Comparisons of our method against state-of-the-art approaches on the Sintel [42] and KITTI 2015 [45] online benchmarks. The EPE and Fl-all are employed as the primary evaluation metrics in this comparison. The notation ‘C + T + S + K + H’ refers to models trained using the combined datasets of FlyingChairs [21], FlyingThings [63], Sintel, KITTI, and HD1K [64]. Results marked with an asterisk (*) indicate that a warm-start strategy was applied. For ease of comparison, the best-performing results are highlighted in bold.

Training	Method	Sintel (Test) ↓		KITTI-15 (Test) ↓
		Clean	Final	Fl-All (%)
C + T + S + K + H	PWC-Net+ [32]	3.45	4.60	7.72
	HD3 [68]	4.79	4.67	6.55
	VCN [25]	2.81	4.40	6.30
	MaskFlowNet [27]	2.52	4.17	6.10
	LiteFlowNet2 [28]	3.48	4.69	7.74
	ScopeFlow [70]	3.59	4.10	6.82
	DICL-FLow [69]	2.12	3.44	6.31
	RAFT [26]	1.61 *	2.86 *	5.10
	Flow1D [34]	2.24	3.81	6.27
	SCV [36]	1.72	3.60	6.17
	GMA [29]	1.39 *	2.47 *	5.15
	Separable Flow [35]	1.50	2.67	4.64
	OCTC [38]	1.82	3.09	4.72
	GMFlow [62]	1.74	2.90	9.32
	GMFlowNet [46]	1.39	2.65	4.79
	MFCFlow [71]	1.49	2.58	5.00
	RFPM [72]	1.41	2.90	4.79
	AGFlow [43]	1.43 *	2.47 *	4.89
	CRAFT [37]	1.45 *	2.42 *	4.79
	DIP [30]	1.67	3.22	**4.21**
	**Ours**	1.38 *	2.76 *	4.75

The improvement in performance highlights HMAFlow’s superior ability to capture local structural features and preserve sharper, more accurate boundaries and contours in optical flow estimation. As shown in Table 2, HMAFlow significantly improves upon RAFT’s performance, reducing the EPE by 14.2% on the clean pass (from 1.61 to 1.38) and by 3.4% on the final pass (from 2.86 to 2.76). To further investigate the source of these improvements, we compare HMAFlow’s performance with several well-known methods [26,29,37,43,62] across different metrics, such as all-pixels, occluded areas, and non-occluded areas, on the Sintel test set. The results are presented in Table 3, showing that while HMAFlow achieves the best performance on the clean pass, it struggles in occluded regions. On the final pass, although our model does not yield the best results, it remains competitive. These findings suggest that HMAFlow may face challenges in handling occluded areas, which could be an avenue for further improvements.

KITTI-15 benchmark. We also conduct a thorough evaluation of HMAFlow on the KITTI 2015 [45] benchmark to assess its performance in real-world scenarios. To ensure a fair comparison with existing methods [26,29], we follow the established ‘C + T + S + K + H’ training setting, which combines multiple datasets, including FlyingChairs, FlyingThings, Sintel, KITTI, and HD1K. The rightmost column of Table 2 presents the quantitative results in terms of the Fl-all (%) metric, which measures the percentage of outlier pixels. HMAFlow achieves a highly competitive Fl-all score of 4.75, surpassing the baseline method RAFT by 6.8%. Although it falls slightly short of the top-performing method on KITTI, this is likely due to the inherent domain differences and the relatively small size of the KITTI dataset, which includes only 200 image pairs—insufficient to train a high-quality model in comparison to larger datasets.

**Figure 8:**
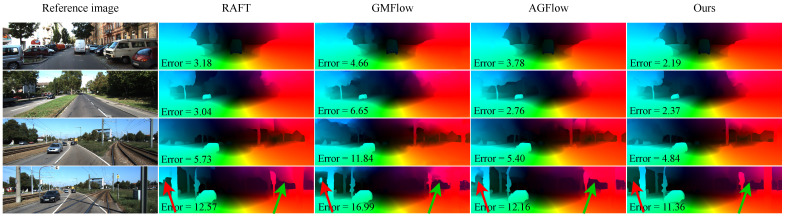
Visual comparisons on the KITTI 2015 [45] test dataset. We evaluate our proposed method, HMAFlow, against three other prominent algorithms: RAFT [26], GMFlow [62], and AGFlow [43], all applied to this realistic driving dataset. In the figure, from left to right, each column represents the input image, RAFT inference, GMFlow inference, AGFlow inference, and our model inference, respectively. When comparing performance using the Fl-all metric, HMAFlow consistently outperforms the other three methods across various scenarios. For instance, as illustrated in the last-row view, our method excels at effectively separating the foreground object from the background sky, thereby demonstrating the superiority and enhanced capabilities of HMAFlow in challenging visual contexts.

To further illustrate the performance improvements, we provide visual comparisons of the estimated optical flow on several sample images from the KITTI test set, as shown in Figure 8. Since we could not find the evaluation results of GMA [29] on the KITTI online benchmark, we compare our method with RAFT [26], GMFlow [62], and AGFlow [43]. These examples highlight HMAFlow’s ability to accurately capture fine details and local structures, which is particularly evident in challenging homogeneous or textureless regions. For instance, in the last row of Figure 8, our model successfully distinguishes utility poles from the sky, producing clear and accurate flow estimates. In contrast, other methods generate blurry and incorrect predictions, failing to differentiate between objects. This visual evidence further underscores the effectiveness of the novel modules introduced in HMAFlow.

In addition to the general Fl-all metric, we also compare HMAFlow with several competitive methods across specific evaluation categories, including the Fl-fg (foreground) and Fl-bg (background) metrics for both all pixels (All) and non-occlusion pixels (Noc). The detailed comparison results are presented in Table 4. Under both the all-pixel and non-occlusion settings, HMAFlow achieves the best overall scores in the Fl-all, Fl-fg, and Fl-bg metrics, outperforming other methods in most cases. The only exception is in the Fl-fg metric under the all-pixels setting, where HMAFlow performs slightly worse than CRAFT [37]. Nonetheless, these results demonstrate that HMAFlow generalizes well to real-world datasets, effectively handling complex scenes with high accuracy and robustness, thereby further reinforcing its effectiveness and reliability in optical flow estimation tasks.

### 4.3. Timing, Parameter, and Accuracy

Building upon the RAFT framework, our model introduces novel modules and additional parameters. To evaluate the feasibility and efficiency of our approach, we conduct a computational complexity analysis comparing our method with other state-of-the-art techniques. Similar to the generalization training process, HMAFlow is trained on the FlyingChairs [21] and FlyingThings [63] datasets. The trained model is then evaluated on the training sets of Sintel [42] and KITTI 2015 [45], while evaluation results for other SOTA methods are obtained from their respective publications. All experiments are conducted in a PyTorch and Python environment using an NVIDIA 3090 GPU, with the parameter count and average inference time recorded for each model. The comparison results are presented in Table 5. We refer to our adjusted baseline model as Baseline (d). The primary reason for the increase in our model’s parameter count is the dimensionality expansion of the output features from the feature encoder and the contextual features input to the GRU network, which was increased from 256 to 384.

We test each model on videos with resolutions of 480×1024 and 1080 p (1088×1920), using millions (M) as the unit for parameter count and seconds (s) as the unit for average inference time per frame. Additionally, we evaluate the real-time performance of each model on videos with a resolution of 480×1024. Among these methods, FlowNet2 [23] has the highest number of parameters, while EMD-S [44] has the fewest (4.5 M). PWC-Net [22] achieves the shortest inference time per frame (0.013 s and 0.046 s), whereas CRAFT [37] records the longest. Our model ranks in the middle in terms of both parameter count and average inference time per frame. The model’s average inference time per frame is not strictly proportional to its parameter count, as the design of the internal architecture also significantly affects runtime. Although EMD-S and PWC-Net perform best in terms of parameter count and inference time, respectively, both exhibit relatively low accuracy. Our model achieves the best performance on the Sintel dataset, while DIP [30] obtains the best results on the KITTI dataset. Although DIP has fewer parameters, its average inference time per frame is longer than that of our model. Compared to RAFT, although our model introduces more parameters, its average inference time remains within an acceptable range. In comparison to GMA [29] and CRAFT, which also utilize attention mechanisms, our model demonstrates highly competitive inference efficiency. In terms of real-time performance evaluation, our model achieves 37 FPS on videos with a resolution of 480×1024, meeting the requirements for real-time applications. Based on the parameter count and real-time performance evaluation results, our model exhibits relatively low computational complexity.

### 4.4. Flow Inference at Occluded Region

Based on the results in Table 2 and Table 3, we speculate that our model has limited capability in occluded regions and on the final subset. To better understand the model’s shortcomings, we compare the performance of several state-of-the-art methods on occluded areas in both the clean and final passes of the Sintel [42] test dataset. The methods compared include RAFT [26], GMA [29], GMFlow [62], GMFlowNet [46], DIP [30], CRAFT [37], AGFlow [43], and our HMAFlow model. All methods are trained on a combined dataset consisting of FlyingChairs [21], FlyingThings [63], Sintel, KITTI [45], and HD1K [64] using standard training procedures. We record the average occluded EPE for all methods on both the clean and final passes, as well as the EPE for each method across 12 occlusion scenarios. All results are presented in Table 6. ‘EPE Unmatched’ refers to the EPE under the occlusion setting. For each scenario, the best-performing model’s results are highlighted in bold.

**Figure 9:**
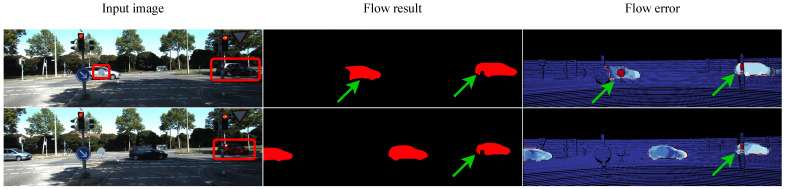
Visual examples of occlusion handling failures. To provide a clearer understanding of our model’s performance in occluded scenarios, we present several failure cases from the KITTI [45] dataset. The first column shows the input image, the second column presents the predicted optical flow, and the third column illustrates the flow error. Red boxes are used to highlight the occluded areas, while green arrows indicate the model’s estimation performance in these regions and mark the incorrect flow predictions (corresponding to the red areas in the flow error subfigure).

Figure 9 illustrates inference failures under occlusion to highlight our model’s limitations. The first column shows input images with occluded regions marked in red boxes, the second column shows the estimated flow, and the third column displays flow errors, with red areas indicating inaccuracies. These examples reveal that the model struggles to estimate flow in occluded regions. Despite this, our model achieves strong performance on both the Sintel and KITTI benchmarks (Table 1, Table 2 and Table 4), and Figure 7 and Figure 8 demonstrate its ability to capture fine object contours. These quantitative and qualitative results confirm the model’s capability in extracting robust features and constructing high-quality cost volumes. However, combined with the above analysis, it is evident that the model remains limited in handling occlusions. To address this, we explore multi-frame flow estimation methods in the subsequent discussion.

On the clean and final sets under occlusion, GMA achieves the best performance (7.963 and 12.501). As shown in Table 2, our model slightly surpasses GMA on the clean pass but falls behind on the final pass, and performs worse overall in occlusion evaluations, indicating difficulties with occluded regions. While RAFT underperforms across all 12 occlusion scenarios, our model achieves the best results in Ambush 3 (12.882, 17.001) and Mountain 2 (0.543, 0.887), showing improved occlusion handling compared to RAFT. The strong performance on the textureless Mountain 2 scene suggests that our model handles low-texture regions well. GMFlow and DIP perform strongly in several scenarios, highlighting their strengths in dealing with occlusion. Unlike these methods, HMAFlow lacks a transformer-based encoder, which may limit its ability to model global dependencies—an aspect we aim to improve in future work.

Our model integrates the HMA module, CSA module, and MCS layer. To evaluate the impact of each component on occlusion handling, we conducted occlusion-specific experiments on the Sintel [42] dataset, with results presented in Table 7. ‘HMA’, ‘MCS’, and ‘CSA’ denote sub-models containing only the respective module, while ‘All’ refers to the complete model. We also evaluated enhanced ‘3-frame’ and ‘5-frame’ variants under five occlusion scenarios: Ambush 1, Bamboo 3, Market 4, Tiger, and Complete frames (referring to all occlusion cases). Bold and underlined values indicate the best and second-best results, respectively. The full model achieved the highest overall performance, with CSA contributing the most to occlusion handling. Nevertheless, occlusion remains a challenging issue. Incorporating multi-frame estimation significantly improves performance, with the ‘5-frame’ model achieving the best results across all scenarios except Tiger.

### 4.5. Evaluation Under More Metrics

We have evaluated our model from different aspects using metrics such as Average EPE, EPE matched, EPE unmatched, Fl-all, Fl-bg, and Fl-fg. The Sintel [42] dataset also provides more evaluation metrics, mainly including d0-10, d10-60, s0-10, and s10-40. d0-10 represents the endpoint error over regions within 10 pixels of the nearest occlusion boundary, while d10-60 covers regions 10 to 60 pixels away from the nearest occlusion boundary. Similarly, s0-10 indicates the endpoint error over regions with motion velocities below 10 pixels per frame, and s10-40 applies to regions with motion velocities between 10 and 40 pixels per frame. We can use these standards to further evaluate and analyze the model’s performance under different error types, which allows for a clearer understanding of the model’s strengths and weaknesses.

We compared our method with RAFT [26], GMA [29], GMFlow [62], GMFlowNet [46], DIP [30], CRAFT [37], and AGFlow [43]. Table 8 reports their results on the clean and final subsets of the Sintel test set under four evaluation criteria, with the best results in bold. Our model achieved the best performance (0.363) under the d10-60 criterion on the clean subset but fell short under d0-10 and both criteria on the final subset, consistent with Table 6’s findings that our model struggles with occlusion. For the clean subset, HMAFlow achieved the best result (0.248) in s0-10 and second-best in s10-40. On the final subset, it achieved the best performance in both s0-10 (0.479) and s10-40 (1.533), showing strong performance across both low-speed and high-speed motion scenarios.

### 4.6. Ablation Studies

To gain deeper insights into the contributions of each component in HMAFlow, we conduct a comprehensive set of ablation studies. In these experiments, we systematically remove one component at a time and train the resulting sub-models on the FlyingChairs and FlyingThings datasets to assess the impact of each module. The number of training iterations, batch size, and learning rate are kept consistent with the standard training settings to ensure a fair comparison. After training, we evaluate the performance of these ablated models on the training sets of the Sintel and KITTI benchmarks. The detailed results of this analysis are summarized in Table 9.

The ablation results confirm that each proposed component in HMAFlow is essential to its overall performance. Removing any individual module significantly impairs the model’s ability to capture fine-grained structural details, leading to noticeable drops in accuracy. When all modules are excluded, the model degrades to the baseline version, which performs poorly on small objects and large motion. In contrast, the full HMAFlow configuration substantially enhances performance in these challenging scenarios, underscoring the effectiveness of the proposed design. The optimal setup includes setting r={4,6,8,10} in the MCS layer, incorporating hierarchical motion features, using a 2×2 convolutional kernel in the HMA module, and applying global positional embedding in the CSA module.

Baseline. For our systematic ablation analysis, we use RAFT as the baseline model, with all components unchanged except for the final feature output dimension *d*, which is set to 384. In the experiments, this modified baseline is referred to as Baseline(d). Correspondingly, the dimensionality of the contextual features fed into the convolutional GRU [33] block is adjusted to 192. These modifications enable the model to process a wider range of contextual information without altering RAFT’s core architecture.
Search strategy. Applying multiple search ranges significantly improves model performance, as shown in Table 3, where increasing the number of search ranges leads to consistent gains. While indexing the two-level base cost volumes with additional ranges keeps the parameter count unchanged, it increases computational complexity. We also compared our multi-scale search strategy with the average pooling method used in RAFT for building hierarchical cost volumes. As shown in Table 9, our approach consistently outperforms average pooling on both datasets, except for nearly identical EPE results on KITTI.
Hierarchical motion. We denote hierarchical motion as “HR Motion” in Table 9. Constructing large cost volumes from high-resolution features offers clear advantages, as these features better capture small targets and local structures. This observation aligns with our results, which demonstrate that hierarchical flow improves performance by preserving fine structural details. The comparisons in Figure 1, Figure 7 and Figure 8 further validate the effectiveness of HR Motion. In contrast, when HR Motion is removed—i.e., when only 1/8 resolution flow is used—the Alignment module becomes redundant.
Correlation self-attention. We further evaluate the contribution of the Correlation Self-Attention (CSA) module to the optimization of 3D cost volume representations. Results show that integrating CSA consistently improves performance—reflected in both EPE and Fl-all metrics—on the Sintel and KITTI 2015 datasets. These findings confirm that capturing global motion relationships via CSA enhances motion representation and leads to more accurate optical flow estimation.
Global position embedding. We denote the global position embedding as “Global PE” in Table 9, referring to our modified positional encoding. Results show that incorporating Global PE into the cost volume improves performance. Compared to the original embedding, Global PE introduces minimal parameters while significantly enhancing robustness against data challenges, offering an efficient design that maintains high performance with low complexity.
Alignment method. Table 9 compares various alignment techniques on two datasets. We test a 2×2 convolution kernel against a 3×3 kernel, average pooling, and max pooling. The 2×2 kernel consistently outperforms the others, likely due to the high-quality 1/4 resolution cost volume, where the larger 3×3 kernel may introduce noise. These results emphasize the importance of carefully selecting alignment strategies, as performance varies across methods.

## 5. Discussion

The aim of this study is to improve the existing RAFT algorithm to address the limitations of current optical flow methods in estimating small, fast-moving objects. Rather than focusing on the application of a specific optical flow algorithm to a particular domain, this work seeks to enhance the overall capability and robustness of optical flow estimation. Optical flow is a well-established and systematic area of fundamental research in computer vision. The Sintel [42] and KITTI [45] datasets are widely used benchmarks for evaluating optical flow algorithms; Sintel is a synthetic dataset, while KITTI is collected from real-world driving scenarios and is extensively used to assess autonomous driving systems. Following previous work, we conduct a comprehensive evaluation of our proposed method, HMAFlow, on both datasets. As described in Section 4, our model demonstrates strong performance across both benchmarks, and the results in Table 8 further show its effectiveness in estimating fast-moving objects.

Based on the experimental results in Table 3 and Table 6, along with the analysis in Section 4.4, it is evident that HMAFlow struggles in occluded regions. Among the compared methods Table 6, GMA [29] achieved the best performance in occlusion evaluation by using cross-attention to aggregate 2D motion and context features into a high-dimensional cost volume. In contrast, our Correlation Self-Attention (CSA) module models global motion associations from a downsampled cost volume, which inevitably loses some information during downsampling. This may limit the CSA module’s ability to capture comprehensive motion cues, explaining HMAFlow’s weaker performance under occlusion. We argue that applying attention mechanisms to high-resolution cost volumes—such as those derived from Vision Transformers (ViTs)—is more effective for modeling global motion than operating on downsampled representations. To address this limitation, we explore multi-frame optical flow estimation, leveraging temporal cues to mitigate occlusion-related performance drops. Specifically, for the *t*-th frame in a video, we compute backward flow with frame t−1 and forward flow with frame t+1 and fuse them using convolution. Likewise, we calculate and fuse backward and forward correlation features. These aggregated flows and correlations are then input into the GRU [33] update block for iterative refinement. Multi-frame estimation can be extended to 3, 5, or more consecutive frames.

Considering inference efficiency, we evaluated 3-frame and 5-frame configurations on the Sintel [42] dataset. As shown in Table 3, ‘2-frame’ denotes the standard two-frame approach, while ‘3-frame’ and ‘5-frame’ refer to multi-frame methods that incorporate temporal context. Further evaluations under specific occlusion scenarios (see Table 7 and Section 4.4) demonstrate that multi-frame strategies significantly improve occlusion handling. The 5-frame configuration yields the best performance on both the clean and final subsets of Sintel and achieves the highest overall accuracy. These findings confirm the effectiveness of multi-frame flow estimation for addressing occlusions. In future work, we plan to explore additional approaches to further enhance the model’s robustness in occluded regions.

## 6. Conclusions

In this study, a novel and effective model, HMAFlow, was designed to capture informative motion features for improving optical flow estimation. HMAFlow integrated the HMA and CSA modules as well as an enhanced MCS layer. These components collectively contributed to producing high-quality cost volumes by leveraging both hierarchical feature correspondences and global motion associations. As a result, HMAFlow achieved state-of-the-art performance compared with other advanced methods on public benchmarks. Notably, HMAFlow considerably improved the prediction accuracy for small, fast-moving objects and preserved fine structural details. We believe that HMAFlow will open new avenues for optical flow research and drive the development of more advanced models. In the future, we plan to focus on improving the model accuracy in occluded regions and optimizing the balance between performance and computational cost for more efficient real-world applications.

## Figures and Tables

**Table 3:** Comparisons using the EPE metric under three different settings: All (all pixels), Noc (non-occluded pixels), and Occ (occluded pixels) on the Sintel [42] test set. The best results for each setting are highlighted in bold for easier comparison. From the comparison results, our model does not achieve optimal performance in optical flow estimation for occluded regions.

Method	Sintel (Clean)			Sintel (Final)		
	All	Noc	Occ	All	Noc	Occ
RAFT [26]	1.61	0.62	9.64	2.86	1.40	14.68
GMA [29]	1.39	0.58	**7.96**	2.47	1.24	**12.50**
GMFlow [62]	1.74	0.65	10.55	2.90	1.31	15.79
CRAFT [37]	1.45	0.61	8.20	**2.42**	**1.16**	12.63
AGFlow [43]	1.43	0.55	8.54	2.47	1.22	12.64
Ours (2-frame)	**1.38**	**0.45**	8.97	2.76	1.22	15.34
Ours (3-frame)	1.32	**0.39**	7.15	2.54	1.10	13.53
Ours (5-frame)	**1.21**	0.41	**6.72**	**2.35**	**1.07**	**12.28**

**Table 4:** Comparison results based on the Fl-bg, Fl-fg, and Fl-all metrics for two specific settings: All (all pixels) and Noc (non-occluded pixels) on the KITTI [45] test benchmark. The best results for each metric are highlighted in bold to facilitate easier comparison among the different methods. Our model demonstrates slightly inferior performance to the best in the Fl-fg metric, while achieving optimal performance consistently across other metrics.

Method	KITTI (All)			KITTI (Noc)		
	Fl-bg	Fl-fg	Fl-All	Fl-bg	Fl-fg	Fl-All
RAFT [26]	4.74	6.87	5.10	2.87	3.98	3.07
GMFlow [62]	9.67	7.57	9.32	3.65	4.46	3.80
CRAFT [37]	4.58	**5.85**	4.79	2.87	3.68	3.02
HMAFlow (Ours)	**4.49**	6.08	**4.75**	**2.62**	**3.33**	**2.75**

**Table 5:** Comparison of parameter quantity and inference time among several state-of-the-art methods. All methods are trained on the FlyingChairs [21] and FlyingThings [63] datasets. The trained models are evaluated on the training sets of the Sintel [42] and KITTI 2015 [45] datasets. The best results are highlighted in bold.

Method	Params (M)	480 × 1024		1088 × 1920	Sintel (Train)		KITTI-15 (Train)	
		Time (s)	FPS	Time (s)	Clean	Final	EPE	Fl-All (%)
FlowNet2 [23]	162.5	0.051	19	0.223	2.02	3.54	10.08	30.0
PWC-Net [22]	9.3	0.013	76	**0.046**	2.55	3.93	10.35	33.7
RAFT [26]	5.3	0.018	55	0.075	1.43	2.71	5.04	17.4
Flow1D [34]	5.7	0.016	62	0.066	1.98	3.27	6.69	22.95
GMA [29]	5.9	0.032	31	0.142	1.30	2.74	4.69	17.1
AGFlow [43]	5.6	0.028	35	0.127	1.31	2.69	4.82	17.0
KPAFlow [39]	5.8	0.039	25	0.176	1.28	2.68	4.46	15.9
CRAFT [37]	6.3	0.062	16	0.281	1.27	2.79	4.88	17.5
DIP [30]	5.1	0.043	23	0.186	1.30	2.82	**4.29**	**13.73**
EMD-S [44]	**4.5**	0.018	55	0.084	1.31	2.67	5.00	17.0
Baseline (d)	8.1	0.020	50	0.091	1.52	2.80	4.68	16.75
HMAFlow (Ours)	10.5	0.027	37	0.125	**1.24**	**2.47**	4.38	14.90

**Table 6:** Analysis of the model’s limitations in occlusion. We compare multiple methods on the Sintel [42] test sets, focusing on inference performance in occluded areas and complex scenarios. We record EPE unmatched results on the clean and final sets, as well as on the 12 occlusion scenarios within each. ‘Complete frames’ refers to the average EPE under occlusion settings of the clean or final set. The best results are highlighted in bold.

Dataset	Scene (EPE Unmatched)	Method							
		RAFT [26]	GMA [29]	GMFlow [62]	GMFlowNet [46]	DIP [30]	CRAFT [37]	AGFlow [43]	Ours
Sintel test (clean)	Complete frames	9.647	**7.963**	10.555	8.486	8.919	8.204	8.541	8.976
	Perturbed Market 3	2.993	2.735	2.329	2.670	**2.200**	2.516	2.773	2.527
	Perturbed Shaman 1	1.790	1.629	1.604	1.696	**1.235**	1.599	1.755	1.670
	Ambush 1	15.458	11.610	**9.055**	11.850	12.538	11.044	13.700	12.142
	Ambush 3	14.824	13.244	13.790	15.063	12.893	14.301	13.214	**12.882**
	Bamboo 3	2.751	2.576	2.501	2.429	**1.908**	2.525	2.671	2.483
	Cave 3	9.188	8.841	**6.490**	8.479	8.298	7.664	8.351	7.066
	Market 1	3.314	2.476	2.323	2.504	**1.630**	2.425	2.468	2.424
	Market 4	16.091	**12.361**	23.741	14.135	16.720	13.337	13.742	16.942
	Mountain 2	0.662	0.705	1.222	1.494	1.582	0.661	0.631	**0.543**
	Temple 1	2.606	2.549	2.256	2.230	**1.891**	2.365	2.535	2.394
	Tiger	4.056	3.914	3.579	3.513	**3.427**	3.774	4.074	3.637
	Wall	7.528	6.602	7.317	**6.261**	6.759	8.185	7.527	6.995
Sintel test (final)	Complete frames	14.680	**12.501**	15.797	13.882	15.485	12.637	12.643	15.341
	Perturbed Market 3	3.382	7.774	**3.146**	3.254	5.588	4.436	3.444	3.590
	Perturbed Shaman 1	1.980	1.779	**1.666**	1.882	1.674	1.706	1.959	1.764
	Ambush 1	38.691	**20.306**	32.352	32.929	34.464	21.254	31.818	34.824
	Ambush 3	20.827	17.504	17.407	17.126	18.941	17.188	19.086	**17.001**
	Bamboo 3	2.943	2.621	2.615	2.702	**2.279**	2.495	2.742	2.522
	Cave 3	11.672	10.927	**8.913**	10.785	11.787	10.700	10.348	9.851
	Market 1	5.050	3.810	4.456	5.532	5.156	**3.662**	3.898	4.261
	Market 4	23.032	21.421	30.755	21.725	27.448	22.768	**19.837**	28.664
	Mountain 2	1.812	2.109	2.240	2.501	2.083	1.674	1.197	**0.887**
	Temple 1	3.399	3.253	3.415	3.372	**2.864**	3.233	3.224	3.050
	Tiger	4.968	4.502	**4.320**	4.928	4.804	4.400	5.028	4.468
	Wall	9.754	9.431	13.170	14.649	9.983	9.573	**7.612**	11.079

**Table 7:** Occlusion evaluation of different sub-models. We evaluate the ability of each module to handle occlusion in various scenarios using the Sintel [42] dataset. ‘Complete frames’ refers to the average EPE measured under occlusion settings for either the clean or final set. The best results are highlighted in bold, and the second-best results are underlined.

Method	Occlusion Scenarios EPE (Clean)					Occlusion Scenarios EPE (Final)				
	Ambush 1	Bamboo 3	Market 4	Tiger	Complete Frames	Ambush 1	Bamboo 3	Market 4	Tiger	Complete Frames
Baseline (d)	14.86	2.71	17.85	4.10	9.31	37.99	2.92	25.68	4.72	16.24
HMA	13.25	2.53	16.96	3.72	9.18	36.52	2.75	26.08	4.58	15.89
MCS	13.79	2.62	17.25	3.85	9.26	35.83	2.74	28.36	4.64	16.23
CSA	12.51	2.49	**16.43**	3.67	9.11	35.08	2.62	**24.85**	4.53	15.67
All	**12.14**	**2.48**	16.94	**3.63**	**8.97**	**34.82**	**2.52**	28.66	**4.46**	**15.34**
3-frame	12.01	2.35	15.77	3.41	7.15	34.16	2.50	24.39	**4.13**	13.53
5-frame	**11.48**	**2.19**	**15.28**	**3.22**	**6.72**	**33.52**	**2.36**	**23.84**	4.25	**12.28**

**Table 8:** Evaluation under the additional metrics of the Sintel [42] test dataset. The Sintel dataset provides additional evaluation metrics, primarily including d0-10, d10-60, s0-10, and s10-40. We compare our model with several state-of-the-art methods under these metrics to analyze its performance across different types of errors. The best results are highlighted in bold.

Dataset	Method	Metrics (EPE)			
		d0-10	d10-60	s0-10	s10-40
clean	RAFT [26]	1.621	0.518	0.341	1.036
	GMA [29]	1.537	0.461	0.331	0.963
	GMFlow [62]	1.232	0.568	0.499	0.971
	GMFlowNet [46]	1.275	0.395	0.314	0.991
	DIP [30]	**1.102**	0.407	0.336	**0.754**
	CRAFT [37]	1.574	0.552	0.311	0.991
	AGFlow [43]	1.501	0.452	0.319	0.963
	Ours	1.310	**0.363**	**0.248**	0.837
final	RAFT [26]	3.112	1.133	0.634	1.823
	GMA [29]	2.863	1.057	0.566	1.817
	GMFlow [62]	**2.491**	1.043	0.714	1.659
	GMFlowNet [46]	2.818	1.050	0.699	1.784
	DIP [30]	2.723	1.090	0.571	1.812
	CRAFT [37]	2.837	1.012	0.538	1.623
	AGFlow [43]	2.892	**0.991**	0.506	1.692
	Ours	2.762	1.088	**0.479**	**1.533**

**Table 9:** Ablation studies. To establish our baseline, we adapt the RAFT [26] model by modifying the dimensions of the output features, increasing them from 256 to 384. All ablated models are trained and evaluated following the same protocol used in the generalization experiments. The final selection of the optimal model is underlined.

Experiments	Method	Sintel		KITTI-15	
		Clean	Final	EPE	Fl-All (%)
RAFT [26]	-	1.43	2.71	5.04	17.4
Baseline (d)	-	1.52	2.80	4.68	16.75
Global PE	No	1.28	2.59	4.43	15.30
	Yes	1.24	2.47	4.38	14.90
Alignment	Conv3×3	1.32	2.54	4.52	15.37
	Conv2×2	1.24	2.47	4.38	14.90
	Average Pooling	1.37	2.63	4.54	15.21
	Max Pooling	1.43	2.59	4.63	15.76
CSA	No	1.33	2.79	4.54	15.61
	Yes	1.24	2.47	4.38	14.90
HR Motion	No	1.36	3.15	4.64	16.67
	Yes	1.24	2.47	4.38	14.90
Search Strategy	r = 4	1.50	2.89	4.48	15.77
	r = 8	1.32	2.67	4.52	15.99
	r = {4, 8}	1.42	2.47	4.26	14.99
	r = {4, 6, 8, 10}	1.24	2.47	4.38	14.90
	Average Pooling	1.35	2.61	4.36	15.24

## Data Availability

Optical Flow Datasets: FlyingChairs available at https://lmb.informatik.uni-freiburg.de/resources/datasets/FlyingChairs.en.html (accessed on 16 September 2023); FlyingThings available at https://lmb.informatik.uni-freiburg.de/resources/datasets/SceneFlowDatasets.en.html (accessed on 16 September 2023); Sintel available at http://sintel.is.tue.mpg.de/ (accessed on 17 September 2023); KITTI 2015 available at https://www.cvlibs.net/datasets/kitti/eval_scene_flow.php?benchmark=flow (accessed on 17 September 2023); HD1k available at http://hci-benchmark.iwr.uni-heidelberg.de/ (accessed on 17 September 2023). Our model’s evaluation results can be found on the Sintel and KITTI 2015 benchmarks, and we have also made our code publicly available on GitHub https://github.com/BooTurbo/HMAFlow (accessed on 2 February 2025).
